# Demographic expansion of an African opportunistic carnivore during the Neolithic revolution

**DOI:** 10.1098/rsbl.2019.0560

**Published:** 2020-01-22

**Authors:** Ahmed Eddine, Rita Gomes Rocha, Noureddine Mostefai, Yamna Karssene, Koen De Smet, José Carlos Brito, Dick Klees, Casten Nowak, Berardino Cocchiararo, Susana Lopes, Peter van der Leer, Raquel Godinho

**Affiliations:** 1 Laboratory of Water Conservatory Management Soil and Forest, Faculty of Sciences of Nature and Life, University of Tlemcen, 13000 Tlemcen, Algeria; 2 Department of Biology and Plant Ecology, University of Setif, 19000 Setif, Algeria; 3 CIBIO/InBIO, Centro de Investigação em Biodiversidade e Recursos Genéticos, Universidade do Porto, Campus de Vairão, 4485-661 Vairão, Portugal; 4 Laboratory of Livestock and Wildlife, Arid Land Institute of Medenine, 4119 Medenine, Tunisia; 5 Society of North African Big Carnivores Stichting, Drabstraat 288, BE-2640 Mortsel, Belgium; 6 Departamento de Biologia, Faculdade de Ciências, Universidade do Porto, 4169-007 Porto, Portugal; 7 Senckenberg Research Institute and Natural History Museum Frankfurt, Conservation Genetics Section, Clamecystraße. 12, 63571 Gelnhausen, Germany; 8 Department of Zoology, University of Johannesburg, PO Box 534, Auckland Park 2006, South Africa

**Keywords:** Holocene Climatic Optimum, *Canis lupaster*, genetic diversity, North Africa, demographic history

## Abstract

The diffusion of Neolithic technology together with the Holocene Climatic Optimum fostered the spread of human settlements and pastoral activities in North Africa, resulting in profound and enduring consequences for the dynamics of species, communities and landscapes. Here, we investigate the demographic history of the African wolf (*Canis lupaster*), a recently recognized canid species, to understand if demographic trends of this generalist and opportunistic carnivore reflect the increase in food availability that emerged after the arrival of the Neolithic economy in North Africa. We screened nuclear and mitochondrial DNA in samples collected throughout Algeria and Tunisia, and implemented coalescent approaches to estimate the variation of effective population sizes from present to ancestral time. We have found consistent evidence supporting the hypothesis that the African wolf population experienced a meaningful expansion concurring with a period of rapid population expansion of domesticates linked to the advent of agricultural practices.

## 1. Introduction

The Neolithic innovations following the domestication of plants and animals have dramatically changed the Mediterranean landscape. The beginning of this impact dates back to approximately 12 000 years BP in the eastern Mediterranean, from where it expanded westwards during the following millennia [1]. The advent of a productive economy, based on farming and the use of domesticated resources, provided the framework for an increase in food availability resulting in rapid human population growth [2,3]. In North Africa, the diffusion of earlier Neolithic technology arrived approximately 9000–7000 years BP [1,4], during the Holocene Climatic Optimum, when a marked climatic shift changed arid desert conditions into savannah-like environments, fostering the establishment of human settlements and the regional development of pastoral activities [5]. The combination of human-induced changes and climate dynamics in North Africa had profound and enduring consequences for the distribution and dynamics of species, communities and landscapes. Notwithstanding negative impacts on biodiversity [6–9], the presence of humans may create advantages for species with the ability to exploit anthropogenic habitats. Several mammal carnivores, for instance, tend to live at higher densities in humanized habitats than in natural ones [10,11]. A variety of opportunities, particularly related to food availability, make human-dominated areas an attractive habitat for opportunistic carnivores. It is thus expected that the Neolithic human population growth had a positive impact on opportunistic wild carnivores that show a propensity for living in cultural landscapes.

The African wolf (*Canis lupaster*), recently recognized as a distinct species of canid [12], is widely distributed across Northern and Eastern Africa [13–15]. Owing to its habitat plasticity and opportunistic feeding habits it occurs in a wide variety of habitats from forest to arid ecosystems, including the vicinity of urban areas [16–19]. Positive relationships between African wolf and anthropogenic areas have been observed [20], including feeding on both wild prey and livestock, and consuming organic waste [16,17]. We thus hypothesized that the ability of this species to exploit human-dominated landscapes has been an advantage during the Neolithic revolution.

Little is still known about basic biological and ecological aspects of the African wolf because of its previous misidentification as a golden jackal (*Canis aureus*). Recent phylogenetic studies conducted on the species identified two main populations in Northwestern and in Eastern Africa [12–15,21,22]. The demographic history of the species, however, remains unknown to date. Here, we collected genetic information of the Northwestern African wolf population to investigate its demographic history, and to understand if demographic trends of this generalist and opportunistic carnivore reflect the increase in availability of food and other human-related opportunities that have emerged since the arrival of the Neolithic economy in North Africa. We thus expect to find evidence for (i) historic population expansion of the African wolf during this period and (ii) a high level of genetic diversity and shallow population structure as signs of a large, interconnected population.

## 2. Material and methods

### (a) Sampling and DNA extraction

Sampling was carried out in Algeria across different ecosystems between 2014 and 2016. DNA extraction followed specific protocols for different sample types (for sampling and DNA extraction details, see electronic supplementary material, appendix S1 and table S1).

### (b) Mitochondrial DNA amplification and sequencing

Mitochondrial (mtDNA) control region was amplified using primers DLH and ThrH [23]. Amplifications were performed in a BioRad T100 Thermal Cycler (electronic supplementary material, table S2). PCR products were purified using ExoSap IT^®^ (Affymetrix) and sequenced using DLH primer using the Big-Dye Terminator v. 3.1 Cycle Sequencing protocol (Applied Biosystems). Electropherograms were checked and aligned using GENEIOUS v. 7.1.5 (https://www.geneious.com).

### (c) Microsatellites genotyping

A set of 47 microsatellite loci was amplified in five multiplex reactions for tissue samples following [24] and [25] (electronic supplementary material, table S3). For scat samples, we genotyped a subset of 13 microsatellites previously optimized in three pools following [26]. Four PCR replicas of each marker were accomplished per non-invasive sample. Amplifications were performed in a BioRad T100 Thermal Cycler (for methodological details, see electronic supplementary material, appendix S1). Amplification products were separated on the ABI 3130xl Genetic Analyser (AB Applied Biosystems) and alleles were scored by comparison to the GeneScan™ 500 LIZ size standard using GENEMAPPER v. 4.1 (Applied Biosystems), and manually checked to control automatic binning.

### (d) Diversity and genetic structure

Mitochondrial diversity was assessed using sequences generated in this study (*n* = 22), and then together with 46 sequences from Algeria and Tunisia retrieved from previous works [14,26]. Diversity indices were assessed using DnaSP 5 [27]. Intraspecific genetic distances were estimated in MEGA 7 [28] using the *p*-distance model. Phylogeographic relationships among the different mtDNA haplotypes were estimated using the median-joining (MJ) network algorithm [29] implemented in PopART [30].

The 47 microsatellite dataset was evaluated for deviations from Hardy–Weinberg (HW) equilibrium using GENALEX v. 6.5 [31], and loci with significant departure from expectations after Bonferroni correction were excluded from the subsequent analysis. Genetic diversity was estimated separately for the dataset of Algeria (*n* = 18), and for the subset of 13 microsatellites in Algeria (*n* = 18 + 2 genotypes from non-invasive samples) and Tunisia (*n* = 27), the latter previously generated in our laboratory [26]. Diversity measures were calculated using GENALEX v. 6.5 [31]. Population structure was tested using the Bayesian clustering approach implemented in STRUCTURE 2.3.4 [32] (for details, see electronic supplementary material, appendix S1) and a principal component analysis (PCA) implemented in the *adegenet* package [33].

Isolation by distance (IBD) was evaluated through Mantel tests implemented in GENALEX v. 6.5 for both molecular markers. The same software was used to test population structure between the two sampling areas (Algeria and Tunisia) through an analysis of molecular variance.

### (e) Demographic analysis

Demographic history of the African wolf was inferred using mitochondrial and microsatellite loci separately, compiling data from Algeria and Tunisia in a single dataset.

For mtDNA, we estimated mismatch distributions and Harpending's raggedness statistics [34], and tested deviation from neutrality through Tajima's D [35] and Fu's Fs [36] statistics, using DnaSP 5 [27]. Smooth and unimodal mismatch distributions, non-significant Harpending's raggedness statistics [34], and significant negative values (*p*-value < 0.05) of Tajima's D and Fu's Fs were taken as evidencing a scenario of demographic expansion. Past population dynamic trend was inferred using Extended Bayesian Skyline Plot (EBSP) implemented in BEAST v. 2.3.2 [37]. We used the strict clock, an evolutionary rate of 5.48% per million years estimated for canids [38] and previously used in the African wolf [14], and HKI + G as the best model of nucleotide substitution as selected in MrModeltest2.3 [39] (electronic supplementary material, appendix S1).

For microsatellite loci, we estimated the variation of effective population sizes (Ne) from present to ancestral time with a coalescent approach using the method VarEff [40] implemented in an R package. The method uses an approximate likelihood of the distribution of distance frequencies between alleles in a Monte Carlo Markov Chain framework [40] (electronic supplementary material, appendix S1). To rule out any possibility of population structure affecting demographic inference [41–43], we additionally performed analyses for Algerian and Tunisian datasets separately (electronic supplementary material, appendix S1).

## 3. Results

### (a) Genetic diversity and structure

**Figure 1.**
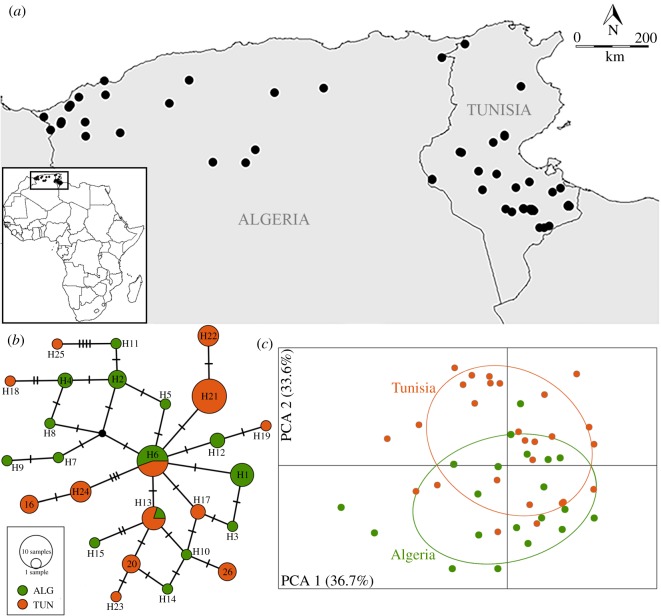
(*a*) Distribution of African wolf samples. (*b*) MJ network based on mtDNA control region, depicting relationships of African wolf haplotypes from Algeria and Tunisia. Dashes in the branches correspond to nucleotide substitutions. (*c*) Principal components analysis (PCA) using 47 African wolf individuals from the Northwestern population analysed for 13 microsatellites; ovals are 95% inertia ellipses for Algerian and Tunisian sampling sites.

A 369 bp fragment of the mtDNA was obtained for 22 African wolves in Algeria, yielding a total of 12 haplotypes with nine segregating sites and high haplotype (Hd) and nucleotide (*π*) diversities (electronic supplementary material, table S4). After merging our dataset with 46 available sequences from Algeria and Tunisia and trimming the fragment to 223 bp, we observed 26 haplotypes with 21 segregating sites (electronic supplementary material, table S4–S5). Ten haplotypes were found for the first time in our study. Two haplotypes (H6 and H13) were shared among Algeria and Tunisia (figure 1). Average intraspecific genetic distance was low (1.7 ± 0.3% sequence divergence). The MJ network exhibits a star-like configuration with a central haplotype (H6) shared between countries. No obvious geographical structure of genetic diversity is revealed by the MJ network (figure 1).

Thirty-eight microsatellite loci [44] showed no deviations from HW expectations in Algeria and were used for the diversity assessment. The African wolf in Algeria exhibited high levels of diversity for all measured indices (electronic supplementary material, table S4). No signal of population structuring was uncovered in African wolves for the different analyses performed (figure 1; electronic supplementary material, figure S1 and S2 and table S6).

We found no correlation between genetic and geographical distances for mtDNA (*R* = 0.075, *p* = 0.09), but a positive correlation was observed for the microsatellite dataset (*R* = 0.149, *p* = 0.004) suggesting some degree of IBD, which is expected in a panmictic population with a very wide range and limited dispersal (electronic supplementary material, table S7). This is also partially evidenced in the PCA (PC2 = 33.6%, figure 1*c*).

### (b) Demographic history

**Figure 2.**
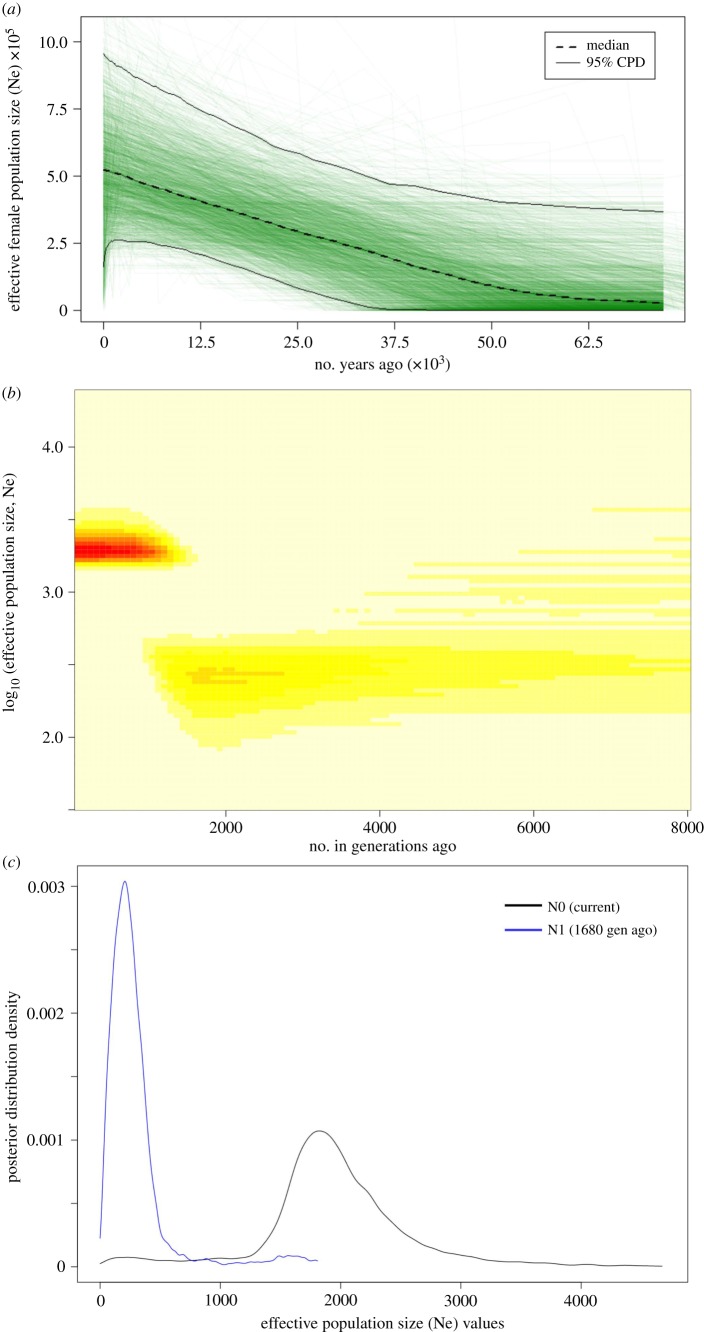
Demographic analysis of the Northwestern African wolf population inferred in BEAST2 and VarEff showing a clear signature of demographic expansion. (*a*) Extended Bayesian Skyline Plot (EBSP) with dashed curve indicating changes in effective female population size (Ne), green lines indicating individual population trajectories and solid curves representing upper and lower 95% highest posterior density (HPD) interval. (*b*) Kernel density of the posterior distribution of the effective population size (Ne) over time. (*c*) Posterior density distribution of Ne at present and at 1680 generations ago, showing non-overlapping of estimates.

We found evidence for a population expansion both in mtDNA and microsatellites. For mtDNA, mismatch distribution showed a smooth and unimodal curve (electronic supplementary material, figure S3), and the observed raggedness value was not significantly different from that expected in expanding populations (*r* = 0.022, *p*-value = 0.06). Both tests of neutrality showed negative values, indicating deviations from the expectations under the mutation–drift equilibrium. Tajima's D indicated negative but non-significant deviations (*D* = −0.878, *p*-value > 0.10), while the Fu's Fs value was negative and significant (Fs = −15.6, *p*-value = 0.0), supporting population expansion. The EBSP supported a population increase to the present, as indicated by the slope of the median effective female population size and the posterior density favouring one population change at *ca* 50 kya (figure 2; electronic supplementary material, figure S3). However, constant population size and two population changes across time were not excluded either (electronic supplementary material, figure S3).

The coalescent approach used to estimate variation in effective population size based on microsatellites detected a pronounced signature of population expansion with non-overlapping distributions of present and past Ne (figure 2). The expansion happened between 960 and 1680 generations in the past, corresponding to the time interval between 3840 and 6720 years BP (figure 2; electronic supplementary material, table S8). The same expansion event was also revealed when Algerian and Tunisian datasets were analysed separately, supporting that this demographic signature is not a result of population sub-structuring (electronic supplementary material, figure S4).

## 4. Discussion

We found consistent evidence supporting our hypothesis that the Northwestern African wolf population experienced a meaningful expansion concurring with a period of rapid population expansion of cattle and other domesticates linked to the advent of agricultural practices during the Neolithic revolution [45,46]. The star-like configuration of MJ network, unimodal mismatch distribution and significant negative neutrality test (Fu's Fs) in mtDNA fit the historical population expansion model. However, further demographic inference using EBSP in mtDNA was equivocal. Although favouring one population change at *ca* 50 kya, constant population size and two population changes across time were not excluded in EBSP. Timing estimates for the expansion detected using mtDNA or microsatellites do not overlap, the latter supporting a clear signature of demographic expansion at approximately 6720–3840 years BP by the end of the wet Holocene Climatic Optimum [47,48]. This resulted in a 7.0-fold increase in the effective population size after which the species demography remained stable.

Discrepancies in inferring demographic histories using different molecular markers are not uncommon [46,49,50], and may be owing to different inheritance mode and/or different evolutionary rates, but also owing to meaningful constraints of using a single short mitochondrial fragment in these analyses [51,52]. For example, different studies on Cape buffalo showed apparently contradictory results in timing and population change [53,54]. These discrepancies were only solved using mitogenomic data, which revealed a two-phased demographic history with a Pleistocene expansion followed by Holocene decline [46]. Similarly, we cannot exclude the hypothesis that the African wolf experienced two population expansions, the first during the Late Pleistocene (*ca* 50 kya) and a second one in the Holocene (approx. 6720–3840 years BP), as detected in mtDNA and microsatellites, respectively. This should be further investigated using additional molecular tools.

Favourable climate change during the Late Pleistocene [55,56] triggered the expansion of wild species [46,57–59] and humans [60], and may have favoured an African wolf expansion. Interestingly, the Holocene African wolf expansion is deeply contrasting with the demographic scenario for other wild species. Mid-Holocene in Africa has been essentially associated with herbivore population declines, as reported for African elephant [61], Cape buffalo [46,53] and common hippopotamus [57], though evidence of range expansion for African lion may be synchronic [62]. Contrasting guild resilience to humans between herbivores and carnivores in the Holocene was explained by differential hunting pressure [63].

The Holocene African wolf expansion detected using microsatellites is remarkably concurrent with African cattle demographic history with pronounced population expansion approximately 5000 years BP [46]. The increasing husbandry of domesticated cattle, sheep and goats [5,64,65] likely represented an unforeseen increase in prey availability for opportunistic carnivores. Selection for tameness during domestication, including the loss of anti-predator behaviours, may have also favoured livestock predation [66,67]. Within carnivore species, population density is typically positively correlated with prey biomass and the number of carnivores supported on given biomass of prey increases with decreasing body size [68]. African wolves are medium-sized carnivores, which supports that higher availability of prey biomass could have fostered a demographic growth of the species.

Although signatures of human-driven demographic processes are well reported in the literature, there is little evidence of favourable historical coexistence of post-Neolithic humans and wild mammals in Africa. Here, we provide evidence of population expansion of an opportunistic species during the Neolithic human revolution, which is possibly related to the increase of human-related resources, such as food availability. Surprisingly, this idea has been little explored.

## Supplementary Material

Supplementary Material

## Data accessibility

DNA sequences: GenBank accession numbers MK659615–MK659624. Microsatellite genotypes: Open Science. Framework doi:10.17605/OSF.IO/YP8CZ.
