# Supplementary material for: Demographic expansion of an African opportunistic carnivore during the Neolithic revolution
Source: Biol Lett. 2020 Jan 22;16(1):20190560. doi: 10.1098/rsbl.2019.0560 (PMC7013491; doi:10.1098/rsbl.2019.0560)
Supplement: Supplementary Material [file rsbl20190560supp1.pdf]

Electronic supplementary material

Demographic expansion of an African opportunistic carnivore during the Neolithic revolution

Ahmed Eddine*, Rita Gomes Rocha*, Noureddine Mostefai, Yamna Karssene, Koen De Smet, José Carlos Brito, Dick Klees, Casten Nowak, Berardino Cocchiararo, Susana Lopes, Peter van der Leer & Raquel Godinho

*These authors contributed equally to this study

Appendix S1

Materials and Methods: details on sample collection, laboratory procedures, data analysis

(a) Sampling and DNA extraction

Sampling was carried out in Algeria across different ecosystems (forest, steppe and desert) between April 2014 and July 2016. It comprised a total of 22 tissue and hair samples from road-kills and poached individuals and three scats (electronic supplementary material, table S1). All samples were preserved in 96% ethanol immediately after collection and then at -20ºC until DNA extraction. The geographical location of each sample was GPS recorded.

DNA extraction of tissue and hair samples was performed using the Genomic DNA Minipreps Tissue Kit (EASY SPIN) following manufacturer's instructions. DNA isolation from scat samples was performed using the GuSCN/silica method of [1]. Handling of non-invasive samples was performed in dedicated laboratory. Negative controls were included throughout the procedures to monitor possible contamination.

(b) Mitochondrial DNA amplification and sequencing

Mitochondrial (mtDNA) control region was amplified using primers DLH and ThrH [2]. Polymerase chain reactions (PCR) were prepared using 5 µl of the QIAGEN Taq PCR Master Mix, 0.4 µM of each primer, 1 µl of DNA extract and water up to a final volume

of 10 µl. Reactions were performed in a BioRad T100 Thermal Cycler (for thermoprofile see electronic supplementary material, table S2). A negative control was included in each PCR to monitor possible contaminations. PCR products were purified using ExoSap IT® (Affymetrix) following manufacturer instructions, and then sequenced using DLH primer using the Big-Dye Terminator v3.1 Cycle Sequencing protocol (Applied Biosystems). Electropherograms were checked and aligned using GENEIOUS 7.1.5 (https://www.geneious.com). All sequences blasted to African wolf in NCBI GenBank database.

(c) Microsatellites genotyping and individual identification

A set of 47 microsatellite loci was amplified in five multiplex reactions for tissue samples following the methodology proposed by [3] and [4] (for details on markers see electronic supplementary material, table S3). For scat samples, we genotyped a subset of 13 microsatellites previously optimized in three pools by [5] following the methodology of these authors. Four PCR replicas of each marker were accomplished per non-invasive sample. Negative controls were included in all PCR amplifications to monitor possible DNA contamination. PCRs were performed in a BioRad T100 Thermal Cycler in final volume reactions of 10 µl including 5 µl of QIAGEN Multiplex PCR Kit, 1 µl of primer multiplex, 3 µl of H_2O and 1 µl of DNA (2.5 µl of DNA for non-invasive samples). PCR profile was specific for each multiplex and according to previously published information referred to above. Amplification products were separated and detected on the ABI 3130xl Genetic Analyser (AB Applied Biosystems) and alleles were scored by comparison to the GeneScan™ 500 LIZ size standard using GENEMAPPER 4.1 (Applied Biosystems), and manually checked to control automatic binning. Identical genotypes corresponding to the same individual were grouped using GIMLET 1.3.3 [6] and excluded from subsequent analysis.

(d) Diversity and genetic structure

Mitochondrial diversity was assessed using sequences generated in this study (n=22), and then together with 46 sequences from Algeria and Tunisia, respectively, retrieved from previous works [5,7; supplementary material, table S5]. Diversity indices were assessed using DnaSP 5 [8]. Intraspecific genetic distances were estimated in MEGA 7 [9] using *p*-distance model. Phylogeographic relationships among the different mtDNA haplotypes

were estimated using the Median-joining (MJ) network algorithm [10] implemented in PopArt [11].

The 47 microsatellite dataset was evaluated for deviations from Hardy–Weinberg equilibrium (HWE) using GENALEX 6.5 [12], and loci with significant departure from expectations after Bonferroni correction were excluded from the subsequent analysis. Genetic diversity was estimated separately for the dataset in Algeria (n=18), and for the subset of 13 microsatellites in Algerian samples including 2 additional genotypes obtained from non-invasive samples from Algeria, and 27 genotypes from Tunisia [5] generated previously in our lab. Diversity measures were calculated using GENALEX 6.5 [12]. Population structure was tested using the Bayesian clustering approach implemented in STRUCTURE 2.3.4 [13]. Analyses were performed independently 5 times for 10^6 iterations after a burn-in period of $5x10^5$ iterations, using the admixture model with correlated allele frequencies among populations. We tested 1 to 10 clusters (K) without prior population information. Structure Harvester [14] was used to summarize the posterior probabilities of each K over all runs [15]. We carried out a Principal Components Analysis (PCA) using the *Adegenet* package in R [16].

Isolation by Distance was evaluated through Mantel tests for mitochondrial and microsatellite loci separately. Three matrices were built including: i) pairwise genetic distance between individuals for each molecular markers estimated in GENALEX 6.5 and, ii) pairwise geographic distance in kilometers from the latitude and longitude of the sampling sites calculated using Geographic Distance Matrix Generator [17]. The Mantel tests were performed in GENALEX 6.5, with significance determined via 999 permutation tests. The same software was used to test population structure between the two sampling areas (Algeria and Tunisia) through an Analysis of Molecular Variance (AMOVA).

(e) Demographic analysis

Demographic history of the African wolf was inferred using mitochondrial and microsatellite loci separately, compiling data from Algeria and Tunisia in a single dataset.

For mtDNA we estimated mismatch distributions and Harpending's raggedness statistics [18], and tested deviation from neutrality through Tajima's D [19] and Fu's Fs [20] statistics, using DnaSP 5 [8]. Coalescence simulations with 1,000 replicates were applied to determine the p-value of each statistic. Smooth and unimodal mismatch distributions, non-significant Harpending's raggedness statistics [18], and significant

negative values (p-value <0.05) of Tajima's D and Fu's Fs were taken as evidencing a scenario of demographic expansion. Past population dynamics was inferred using Extended Bayesian Skyline Plot (EBSP) implemented in BEAST 2.3.2 [21]. We used the strict clock, an evolutionary rate of 5.48% per million years estimated for canids [22] and previously used in this species [7], and HKI+G as best model of nucleotide substitution as selected in MrModeltest2.3 [23]. Two independent runs of 10^8 generations each and sampled every 10^4 generations were performed. Tracer 1.6 [24] was used to check convergence of the MCMC chains. The Extended Bayesian skyline plot (EBSP) was constructed in R platform (R Core Team, 2018).

For microsatellite loci we estimated the variation of effective population sizes (Ne) from present to ancestral time with a coalescent approach using the method VarEff [25] implemented in a R package. The method uses an approximate likelihood of the distribution of distance frequencies between alleles in a Monte Carlo Markov Chain framework [25]. After several trial runs, the final analyses were conducted using the two phase mutation model assuming a proportion of 0.22 for multi-step mutations [26], a mutation rate of 3.5×10^{-3} [27] and allowing three population size changes (JMAX = 3). Prior for current Ne was set according to estimation on trial runs (NBAR = 1,600). Prior for the number of generations since the origin of the population (GBAR) was set to 8000 generations (equivalent to 32 kya, based on a generation time of 4 years for wolves from [28]) to encompass timing of Neolithic expansion in North Africa. Final run was carried out using 10,000 batches with a length of 10, saved every 10 batches in the MCMC chain and with a burn-in period of 10,000 batches.

(f) Verification of demographic inference results

Demographic inference can be affected by population structure, non-random sampling, lack of information in molecular markers and natural selection [29–31]. In order to rule out obvious confounders and possible batch effect, we performed additional demographic analyses for microsatellites using subsets of our samples per country.

We implemented the same coalescent approach using the method VarEff with the same priors as described above, except for the current Ne which was set to 1,600 and 1,200 for Algeria and Tunisia, respectively, as estimated in trial runs. This coalescent approach confirmed a pronounced signature of population expansion for both Algerian and Tunisian datasets (electronic supplementary material, figure S4). The expansion event estimated separately for each subset of samples is concordant with that estimated

using the combination of Algerian and Tunisian samples. This event happened between 960 and 1,680 generations in the past, corresponding in time to the interval between 3,840 and 6,720 years BP. This supports that the observed signature of population expansion is not a result of Northwestern African wolf population sub-structuring.

Table S1. African wolf samples collected throughout Algeria, including type of sample, geographic location (longitude and latitude), indication of available microsatellite genotypes, mtDNA haplotype code and GenBank accession numbers for the new haplotypes of mtDNA control region.

Sample	Type	Longitude	Latitude	Microsatellites	mtDNA	GenBank
CH04	Scat	9.281255	24.843176	Yes	-	
CH06	Tissue	3.159227	33.790477	Yes	H1	MK659615
CH07	Hair	2.337539	33.797816	Yes	H1	
CH08	Hair	0.016947	34.542942	Yes	H2	
CH09	Scat	-1.477441	34.778433	No	H3	MK659616
CH10	Tissue	-1.454867	34.794498	Yes	H4	MK659617
CH12	Tissue	0.450367	35.142239	Yes	-	
CH13	Tissue	-0.849889	34.454156	Yes	H5	MK659618
CH18	Hair	5.118635	35.65465	Yes	H6	
CH19	Tissue	-1.906344	34.935076	Yes	H7	MK659619
CH20	Tissue	-1.454867	34.822239	Yes	H1	
CH21	Tissue	-0.858917	34.809296	Yes	H4	
CH22	Hair	-0.353265	35.493187	Yes	H8	MK659620
CH24	Scat	9.353495	24.93196	Yes	-	
CH23	Tissue	-1.472927	34.764904	Yes	H1	
CH26	Tissue	-1.725753	34.616928	Yes	H9	MK659621
CH27	Hair	-0.389381	35.837722	Yes	H10	MK659622
CH28	Hair	3.881595	35.544562	Yes	H1	
CH29	Hair	1.741591	35.86015	Yes	H6	
CH30	Hair	1.253995	35.280347	Yes	H6	
CH31	Hair	3.403027	34.113405	Yes	H6	
CH32	Hair	8.080331	36.430028	Yes	H11	MK659623
CH34	Tissue	-1.238159	35.238424	Yes	H2	
CH35	Hair	-1.283305	35.171836	Yes	H12	MK659624
CH36	Tissue	-1.021449	35.430792	Yes	H12	

Table S2. Polymerase Chain Reaction (PCR) thermoprofile for amplification of the mtDNA control region fragment.

Thermocycling profile		
**Temperature**	**Time**	**N cycles**
95ºC	15’	1
95ºC	30’’	
50ºC	30’’	40
72ºC	45’’	
60ºC	10’	1

Table S3. Microsatellite multiplex and PCR thermocycling conditions for the African wolf (multiplexes following [4]; PCR thermocycling adjusted for this study). *loci excluded from the analysis; #loci used to amplify non-invasive samples.

Multiplex	Microsatellites	Dye	PCR profile Temperature	Time	N cycles
			95ºC	15’	1
MS1	AHT132	VIC	95ºC	30’’	
	C27.442	PET	58ºC	45’’	20 (-0.1ºC/cycle)
	FH2010	FAM	72ºC	45’’	
	FH2079	NED	95ºC	30’’	
	PEZ1	FAM	56ºC	45’’	15
	PEZ3#	NED	72ºC	45’’	
	PEZ5*	VIC	95ºC	30’’	
	PEZ8*	XXX	53ºC	45’’	10
			72ºC	45’’	
			60ºC	30’	1
MS2	AHT103*	NED			
	AHT111#	VIC	95ºC	15’	1
	C04.140	PET	95ºC	30’’	
	C09.173	NED	56ºC	45’’	35
	C13.758	FAM	72ºC	45’’	
	C14.866	VIC	95ºC	30’’	
	C20.253#	PET	53ºC	45’’	8
	CPH14	FAM	72ºC	45’’	
	FH2001	FAM	60ºC	30’	1
	VWF#	NED			
MS3	C08.140#	VIC	95ºC	15’	1
	C08.618	VIC	95ºC	30’’	
	C09.474	PET	60ºC	45’’	7 (-0.5ºC/cycle)
	C20.446	NED	72ºC	45’’	
	C22.763*	Xxx	95ºC	30’’	
	CPH02#	NED	57ºC	45’’	22
	CPH05#	FAM	72ºC	45’’	
	CPH09*	NED	95ºC	30’’	
	CXX.459*	VIC	53ºC	45’’	8
	FH2161	NED	72ºC	45’’	
	REN64E19#	FAM	60ºC	30’	1
Thermo Fisher Scientific (Canine Genotypes Panel 2.1 Kit)	AHT121	PET			
	AHT137	VIC			
	AHTh171#	PET			
	AHTh260*	VIC			
	AHTk211*	FAM			
	AHTk253	VIC			
	C22.279#	FAM	98ºC	3’	1
	FH2054#	PET	98ºC	15’’	
	FH2848	NED	60ºC	75’’	40
	INRA21#	VIC	72ºC	45’’	
	INU005	NED	72ºC	5’	1
	INU030*	NED			
	INU055	FAM			
	REN162C04#	PET			
	REN169D01	VIC			
	REN169O18	FAM			
	REN247M23	PET			
	REN54P11	FAM			

Table S4. Genetic diversity of the African wolf based on mitochondrial control region (mtDNA) and microsatellite data for samples collected in Algeria, Tunisia and a combined dataset from Algeria and Tunisia. Information for mtDNA includes number of samples (n), sequence length in base pairs (bp), number of haplotypes (h), segregation sites (S), haplotype (Hd) and nucleotide (π) diversities, standard deviations between parentheses, neutrality tests of Tajima's D and Fu's Fs, and raggedness index r. Information for microsatellites includes number of loci (loci), average number of alleles/locus (Na), observed (Ho) and expected (He) heterozygosities and fixation index (Fis), and standard errors between parentheses. [1]this study only. Statistical significance: * P < 0.05, ** P < 0.01, *** P < 0.001.

	**Algeria+Tunisia**	**Algeria[1]**	**Tunisia**
**mtDNA**			
**N**	68	22	41
**bp**	223	369	223
**H**	26	12	15
**S**	21	9	22
**Hd**	0.944 (0.013)	0.918 (0.040)	0.918 (0.02)
**Π**	0.016 (0.001)	0.011 (0.001)	0.018 (0.005)
**D**	-0.878	-0.166	-0.733
**Fs**	-15.634***	-6.096**	-3.113
**r**	0.022	0.052	0.020*
**Microsatellites**			
**N**	47	18	27
**Loci**	13	38	13
**Na**	9.3 (0.3)	7.7 (0.3)	9.3 (0.4)
**Ho**	0.770 (0.02)	0.715 (0.02)	0.775 (0.02)
**He**	0.830 (0.01)	0.773 (0.01)	0.821 (0.01)
**Fis**	0.071 (0.02)	0.078 (0.02)	0.054 (0.03)

Table S5. Correspondence between mtDNA haplotype code and GenBank accession numbers.

	GenBank accession numbers		
	**This work**	**Karssene et al. 2018**[5]	**Gaubert et al. 2012**[7]
H1	MK659615		
H2			JQ088680
H3	MK659616		
H4	MK659617		
H5	MK659618		
H6			JQ088678
H7	MK659619		
H8	MK659620		
H9	MK659621		
H10	MK659622		
H11	MK659623		
H12	MK659624		
H13			JQ088682
H14			JQ088681
H15			JQ088679
H16		MK392560	
H17		MK392566	
H18		MK392562	
H19		MK392568	
H20		MK392563	
H21		MK392572	
H22		MK392569	
H23		MK392564	
H24		MK392561	
H25		MK392565	
H26		MK392571	

Table S6. Analyses of Molecular Variance (AMOVA) results for both Algeria and Tunisia based on mitochondrial DNA and microsatellites, including degrees of freedom (df) and percentage of variance (%).

	df	% var.
**mtDNA**		
Among populations	1	9
Within populations	67	91
Total	68	100
**Microsatellites**		
Among populations	1	1
Within populations	45	11
Within individuals	47	87
Total	93	100

Table S7. Correlation coefficient (R) between genetic and geographic matrices estimated throughout Mantel test for both Algeria and Tunisia, and for each country separately, including p-value. Significant values are in bold.

	**Algeria + Tunisia**		**Algeria**		**Tunisia**	
**IBD**	**mtDNA**	**microsatellites**	**mtDNA**	**microsatellites**	**mtDNA**	**microsatellites**
R	0.075	0.149	0.124	0.174	0.069	0.194
p-value	0.09	**0.004**	0.09	0.14	0.19	**0.01**

Table S8. Posterior estimates of effective population size (Ne) for the African wolf at different time in the past (240 generations intervals), calculated with VarEff, for both Algeria and Tunisia (ALG+TUN). Time is given in generations. h. mean: harmonic mean; HPD: highest posterior density (intervals).

**Time**	**Effective population size**				
Generations	*h. mean*	*Mode*	*median*	*HPD 5%*	*HPD 95%*
0	1122	1705	1717	519	2474
240	1901	1712	1826	1404	4447
480	1907	1702	1844	1402	4949
720	1751	1723	1823	1047	5001
960	1199	1706	1729	390	4204
1200	609	353	1442	219	2873
1440	314	295	390	122	2020
1680	202	246	295	73	1609
1920	176	231	269	63	1071
2160	174	231	270	62	1096

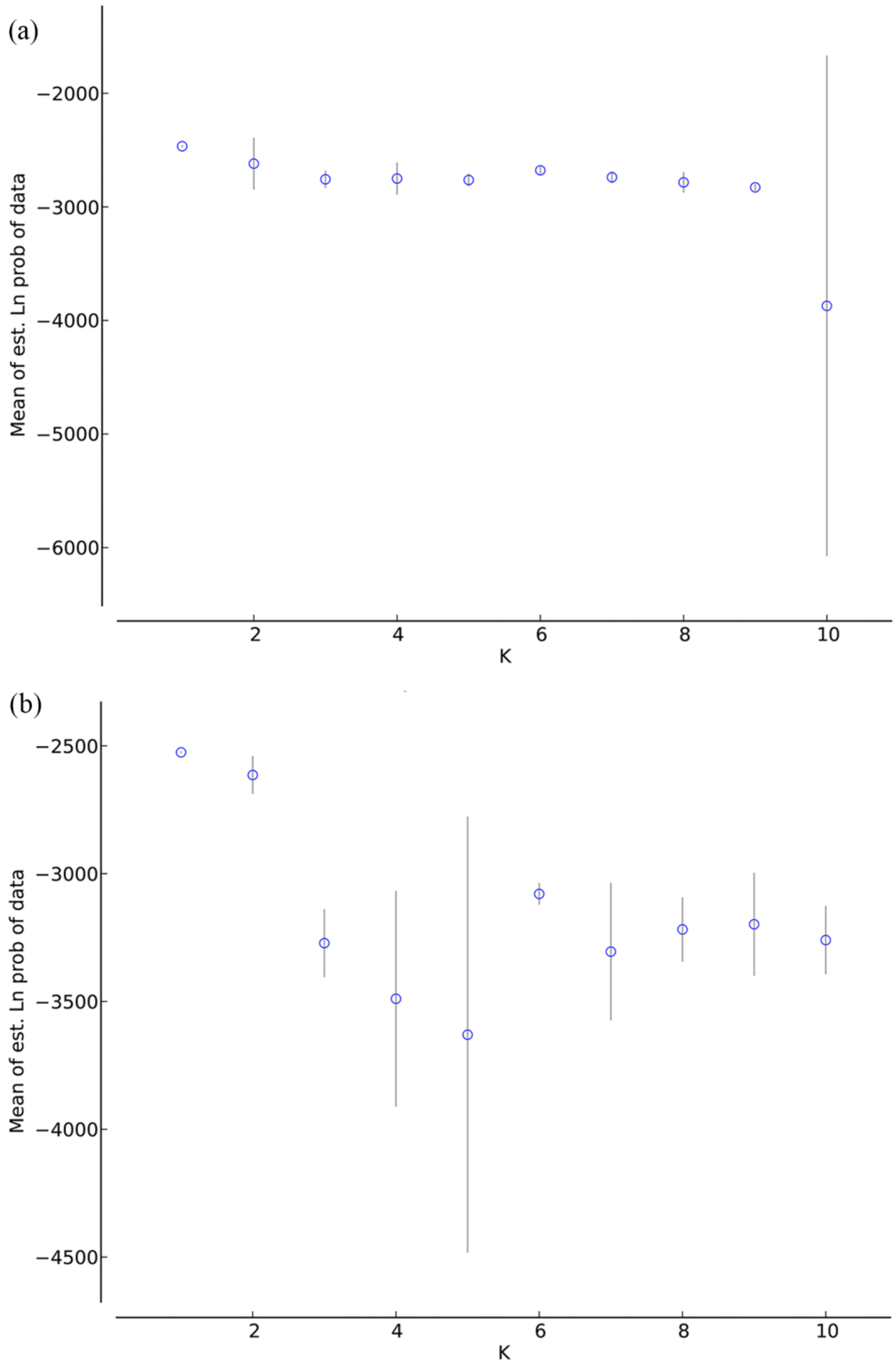

Figure S1. Mean log-likelihood distribution for each cluster (K) and standard deviation bars as summarized through Structure Harvester [14] for (a) Algerian dataset using 38 loci, and (b) Algerian and Tunisian datasets using 13 loci.

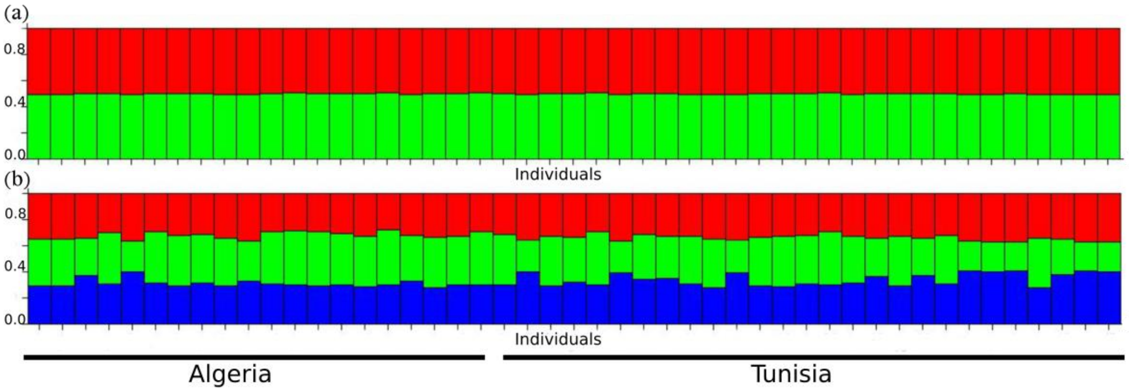

Figure S2. Results of the clustering analysis in STRUCTURE for Algerian and Tunisian datasets using 13 loci. (a) Barplot based on the K=2 and (b) barplot based on the K=3, both showing admixture between individuals. Each vertical bar represents an individual.

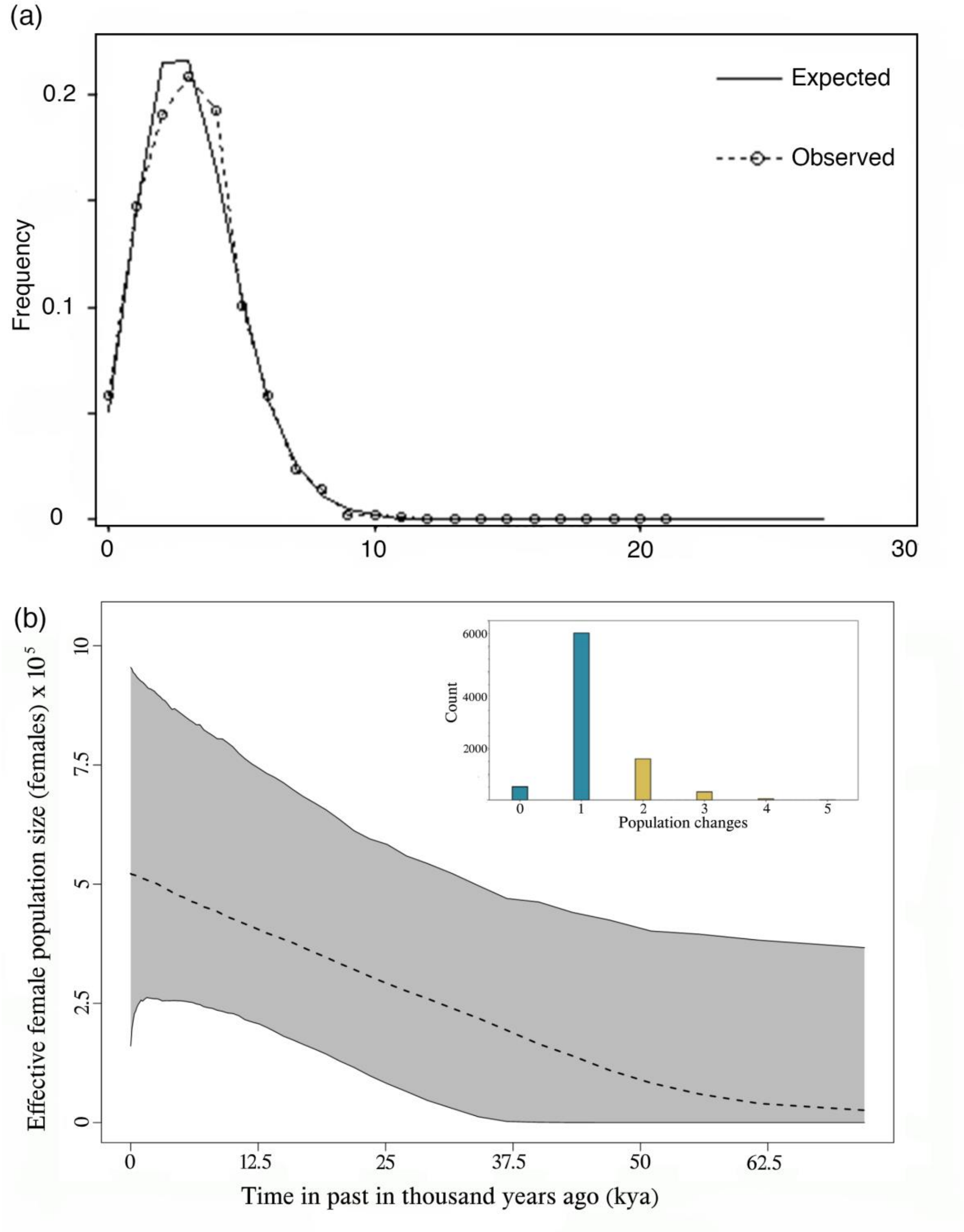

Figure S3. Demographic analysis of the African wolf population from Algeria and Tunisia using 223bp of the mitochondrial control region. (a) Mismatch distribution graph inferred in DNAsp where dashed curve indicates the observed frequency distribution of pairwise differences and solid curve indicates the distribution expected under a population growth–decline model. (b) Extended Bayesian skyline plot inferred in BEAST2, with dashed curve indicating changes in effective population size and shaded region representing 95% highest posterior density (HPD) interval. Insert represent the number of counts per number of population changes (mean=1.2, median=1, 95% HPD=0–2).

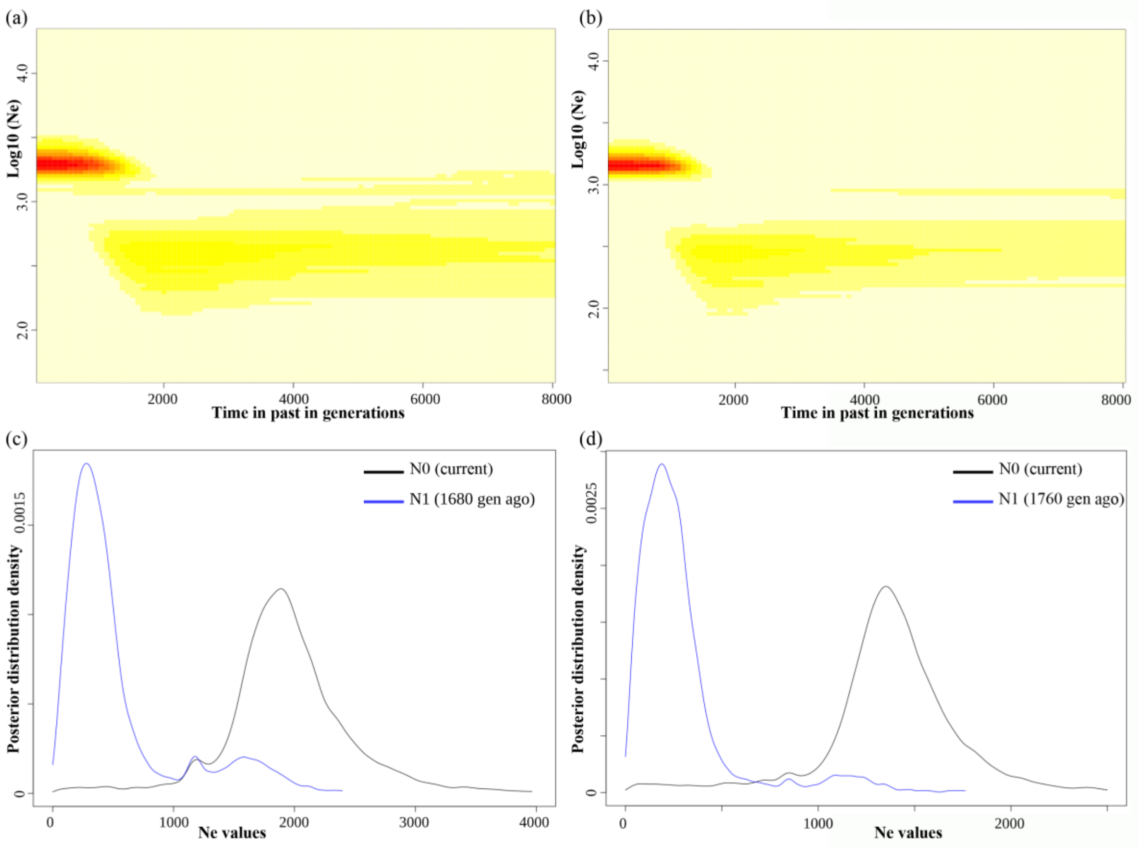

Figure S4. Demographic analysis of the African wolf population from (a and c) Algeria and (b and d) Tunisia inferred in VarEff. Upper panel: Kernel density of the posterior distribution of the effective population size (Ne) over time. Lower panel: Posterior density distribution of Ne at present and at 1,680 generations ago.

References

1. Frantz AC, Pope LC, Carpenter PJ, Roper TJ, Wilson GJ, Delahay RJ, Burke T. 2003 Reliable microsatellite genotyping of the Eurasian badger (Meles meles) using faecal DNA. *Mol. Ecol.* **12**, 1649–1661. (doi:10.1046/j.1365-294X.2003.01848.x)
2. Kocher TD, Thomas WK, Meyer A, Edwards S V, Pääbo S, Villablanca FX, Wilson AC. 1989 Dynamics of mitochondrial DNA evolution in animals: amplification and sequencing with conserved primers. *Proc. Natl. Acad. Sci. U. S. A.* **86**, 6196–200. (doi:10.1073/PNAS.86.16.6196)
3. Godinho R *et al.* 2011 Genetic evidence for multiple events of hybridization between wolves and domestic dogs in the Iberian Peninsula. *Mol. Ecol.* **20**, 5154–5166. (doi:10.1111/j.1365-294X.2011.05345.x)
4. Silva P *et al.* 2018 Cryptic population structure reveals low dispersal in Iberian wolves. *Sci. Rep.*
5. Karssene Y *et al.* 2018 Noninvasive genetic assessment provides evidence of extensive gene flow and possible high movement ability in the African golden wolf. *Mamm. Biol.* **92**, 94–101. (doi:10.1016/j.mambio.2018.05.002)
6. Valière N. 2002 gimlet: a computer program for analysing genetic individual identification data. *Mol. Ecol. Notes* **2**, 377–379.
7. Gaubert P, Bloch C, Benyacoub S, Abdelhamid A, Pagani P, Djagoun CAMS, Couloux A, Dufour S. 2012 Reviving the african wolf canis lupus lupaster in north and west africa: A mitochondrial lineage ranging more than 6,000 km wide. *PLoS One* **7**. (doi:10.1371/journal.pone.0042740)
8. Librado P, Rozas J. 2009 DnaSP v5: A software for comprehensive analysis of DNA polymorphism data. *Bioinformatics* **25**, 1451–1452.
9. Kumar S, Stecher G, Tamura K. 2016 MEGA7: Molecular Evolutionary Genetics Analysis version 7.0. *Mol. Biol. Evol.* **33**, 1870–1874.
10. Bandelt HJ, Forster P, Röhl A. 1999 Median-joining networks for inferring intraspecific phylogenies. *Mol. Biol. Evol.* **16**, 37–48.
11. Leigh JW, Bryant D. 2015 PopART: Full-feature software for haplotype network construction. *Methods Ecol. Evol.* **6**, 1110–1116.
12. Peakall R, Smouse PE. 2012 GenAlEx 6.5: genetic analysis in Excel. Population genetic software for teaching and research--an update. *Bioinformatics* **28**, 2537–2539.

13. Falush D, Stephens M, Pritchard JK. 2003 Inference of Population Structure Using Multilocus Genotype Data: Linked Loci and Correlated Allele Frequencies. *Genetics* **164**, 1567 LP – 1587.
14. Earl DA, VonHoldt BM. 2012 STRUCTURE HARVESTER: a website and program for visualizing STRUCTURE output and implementing the Evanno method. *Conserv. Genet. Resour.* **4**, 359–361.
15. Evanno G, Regnaut S, Goudet J. 2005 Detecting the number of clusters of individuals using the software STRUCTURE: a simulation study. *Mol. Ecol.* **14**, 2611–2620. (doi:10.1111/j.1365-294X.2005.02553.x)
16. Jombart T. 2008 adegenet: a R package for the multivariate analysis of genetic markers. *Bioinformatics* **24**, 1403–1405. (doi:10.1093/bioinformatics/btn129)
17. Ersts PJ. In press. Geographic Distance Matrix Generator(version 1.2.3). *Am. Museum Nat. Hist. Cent. Biodivers. Conserv.* See http://biodiversityinformatics.amnh.org/open_source/gdmg (accessed on 29 April 2019).
18. Harpending HC. 1994 Signature of ancient population growth in a low-resolution mitochondrial DNA mismatch distribution. *Hum. Biol.* **66**, 591–600.
19. Tajima F. 1989 Statistical method for testing the neutral mutation hypothesis by DNA polymorphism. *Genetics* **123**, 585–595.
20. Fu YX. 1997 Statistical tests of neutrality of mutations against population growth, hitchhiking and background selection. *Genetics* **147**, 915–925.
21. Bouckaert R, Heled J, Kühnert D, Vaughan T, Wu C-H, Xie D, Suchard MA, Rambaut A, Drummond AJ. 2014 BEAST 2: A software platform for Bayesian evolutionary analysis. *PLoS Comput. Biol.* **10**, e1003537.
22. Li Q *et al.* 2008 Origin and phylogenetic analysis of Tibetan Mastiff based on the mitochondrial DNA sequence. *J. Genet. Genomics* **35**, 335–340. (doi:10.1016/S1673-8527(08)60049-1)
23. Nylander JAA. 2004 MrModeltest, version 2. Program distributed by the author.
24. Rambaut A, Drummond AJ. 2013 Tracer.
25. Nikolic N, Chevalet C. 2014 Detecting past changes of effective population size. *Evol. Appl.* **7**, 663–681. (doi:10.1111/eva.12170)
26. Peery MZ, Kirby R, Reid BN, Stoelting R, Jonathan N, Robinson S, Va C. 2012 Reliability of genetic bottleneck tests for detecting recent population declines. *Mol. Ecol.* **21**, 3403–3418. (doi:10.1111/j.1365-294X.2012.05635.x)

27. Parra D, García D, Mendez S, Cañon J, Dunner S. 2009 High Mutation Rates in Canine Tetranucleotide Microsatellites : Too Much Risk for Genetic Compatibility Purposes ? *Open Forensic Sci. J.* **2**, 1–5.
28. Mech LD, Barber-meyer SM, Erb J. 2016 Wolf (Canis lupus) Generation Time and Proportion of Current Breeding Females by Age. *PLoS One* **11**, e0156682. (doi:10.1371/journal.pone.0156682)
29. Stiller M *et al.* 2010 Withering away-25,000 years of genetic decline preceded cave bear extinction. *Mol. Biol. Evol.* **27**, 975–978. (doi:10.1093/molbev/msq083)
30. Heller R, Chikhi L, Siegismund HR. 2013 The Confounding Effect of Population Structure on Bayesian Skyline Plot Inferences of Demographic History. *PLoS One* **8**, e62992. (doi:10.1371/journal.pone.0062992)
31. Chikhi L, Sousa VC, Luisi P, Goossens B, Beaumont MA. 2010 The confounding effects of population structure, genetic diversity and the sampling scheme on the detection and quantification of population size changes. *Genetics* **186**, 983–995. (doi:10.1534/genetics.110.118661)
